# *Zingiber Officinale* Roscoe Prevents Cellular Senescence of Myoblasts in Culture and Promotes Muscle Regeneration

**DOI:** 10.1155/2020/1787342

**Published:** 2020-04-29

**Authors:** Nur Fatin Nabilah Mohd Sahardi, Faizul Jaafar, Mariam Firdhaus Mad Nordin, Suzana Makpol

**Affiliations:** ^1^Department of Biochemistry, Faculty of Medicine, Level 17, Preclinical Building, Universiti Kebangsaan Malaysia Medical Centre, Jalan Yaacob Latif, Bandar Tun Razak, Cheras, 56000 Kuala Lumpur, Malaysia; ^2^Department of Chemical Process Engineering, Universiti Teknologi Malaysia (UTM) Kuala Lumpur, Jalan Sultan Yahya Petra, 54100 Kuala Lumpur, Malaysia

## Abstract

**Background:**

Ageing resulted in a progressive loss of muscle mass and strength. Increased oxidative stress in ageing affects the capacity of the myoblast to differentiate leading to impairment of muscle regeneration. *Zingiber officinale* Roscoe (ginger) has potential benefits in reversing muscle ageing due to its antioxidant property. This study aimed to determine the effect of ginger in the prevention of cellular senescence and promotion of muscle regeneration.

**Methods:**

Myoblast cells were cultured into young and senescent state before treated with different concentrations of ginger standardised extracts containing different concentrations of 6-gingerol and 6-shogaol. Analysis on cellular morphology and myogenic purity was carried out besides determination of SA-*β*-galactosidase expression and cell cycle profile. Myoblast differentiation was quantitated by determining the fusion index, maturation index, and myotube size.

**Results:**

Treatment with ginger extracts resulted in improvement of cellular morphology of senescent myoblasts which resembled the morphology of young myoblasts. Our results also showed that ginger treatment caused a significant reduction in SA-*β*-galactosidase expression on senescent myoblasts indicating prevention of cellular senescence, while cell cycle analysis showed a significant increase in the percentage of cells in the G_0_/G_1_ phase and reduction in the S-phase cells. Increased myoblast regenerative capacity was observed as shown by the increased number of nuclei per myotube, fusion index, and maturation index.

**Conclusions:**

Ginger extracts exerted their potency in promoting muscle regeneration as indicated by prevention of cellular senescence and promotion of myoblast regenerative capacity.

## 1. Introduction

Sarcopenia is one of the age-related diseases which is defined as the progressive loss of muscle mass and strength [1]. Sarcopenia begins approximately at the age of 40 years in which muscle mass is predicted to decline about 8% per decade until the age of 70 years [2]. The progressive loss of muscle mass is characterized by the decrement of regenerative capacity of the skeletal muscle involving myoblast cells which can result in impairment of muscle regeneration and difficulty in movement [3].

In the development of the skeletal muscle, satellite cell is a vital component as it will proliferate and differentiate into the myoblast before fuse with another adjacent muscle fibre in order to form a new muscle fibre. As ageing occurs, the capacity of the satellite cell to regenerate is reduced resulting in decreased muscle mass and strength [3]. Several factors including hormonal change, reduction in physical activity, insulin change, increased level of oxidative stress, genetic susceptibility, and nutritional deficiencies may contribute to this age-related disease [4].

Oxidative stress is depicted by the presence of reactive oxygen species (ROS). The level of oxidative stress is increased when the antioxidant level is decreased due to the low antioxidant capacity, impaired antioxidant enzyme activity, and increased ROS production [5]. The increment of oxidative stress which is represented by the overproduction of ROS could lead to oxidative damage of some biological molecules including DNA, protein, carbohydrates, and lipid. This further causes alteration of DNA transcription, loss of DNA repair capacity, lipid peroxidation, mitochondrial dysfunction, alteration of cell growth and differentiation, induction of mutation, and apoptosis [6–8].

Ginger (*Zingiber officinale* Roscoe) is one of the potential herbs that can be used in scavenging free radicals produced during oxidative stress and inflammation [9]. Ginger not only possesses antioxidant properties but also displays anticancer [10], antibacterial [11], antidiabetic [12], and anti-inflammatory [13] properties. Several bioactive compounds including 6-gingerol, 6-shogaol, 10-gingerol, gingerdiones, gingerdiols, paradols, 6-dehydrogingerols, 5-acetoxy-6-gingerol, 3,5-diacetoxy-6-gingerdioal, and 12-gingerol [14] have been characterised in ginger. However, the most abundant active compounds in ginger are 6-gingerol and 6-shogaol. The antioxidant effects in 6-gingerol and 6-shogaol have been shown on various types of diseases caused by oxidative stress, such as cardiovascular disease [15], cancer [16], and diabetes [17]. A previous *in vitro* study showed that treatment of ginger extract on liver cancer cell line removed the superoxide radicals and hydrogen peroxide by replacing the function of glutathione peroxidase (GPx), superoxide dismutase (SOD), and catalase (CAT) [16].

Hosseinzadeh et al. reported that the ginger extract which contains 6-gingerol and 6-shogaol reduced the production of ROS and lipid peroxidation as well as induced the expression of the antioxidant enzyme, namely, CAT, SOD, glutathione peroxidase-1 (GPx1), glutathione peroxidase-3 (GPx3), and glutathione peroxidase-4 (GPx4), in the human chondrocyte model [18]. A previous study performed by Lee et al. found that 6-gingerol suppressed the expression of *β*-amyloid, increased the expression of antioxidant enzyme, and restored glutathione level in Alzheimer's disease [19]. 6-Shogaol also displayed a positive result in Parkinson disease by protecting dopaminergic neurons against MPTP^−^- and MPP^+^-induced neurotoxicity as well as inhibiting the release of nitric oxide and reduced the expression of inducible nitric oxide synthase (iNOS) [20, 21].

However, currently, no study has been conducted to determine the effect of 6-gingerol and 6-shogoal in ginger extracts on cellular senescence and myoblast differentiation. Thus, this study aimed to elucidate the effect of 6-gingerol and 6-shogaol in standardised ginger extracts in the prevention of cellular senescence and promotion of myoblast differentiation in the culture.

## 2. Materials and Methods

### 2.1. Cell Culture and Replicative Senescence

Primary human myoblasts (human skeletal muscle myoblast; HSMM) which were derived from a 17-year-old female were purchased from Lonza (Walkersville, MD, USA). Myoblast cells were maintained in the growth medium, skeletal muscle growth media-2 (SkGM-2 medium) that contained the skeletal muscle basal medium (SkBM), and complete culture media (CCM). SkGM-2 consists of human epidermal growth factor (HeGF), L-glutamine, dexamethasone, foetal bovine serum (FBS), and gentamicin/amphotericin B (Lonza, Walkersville, MD, USA). When cell populations reached 70–80% of confluency, cells were trypsinised. The medium was warmed to 37°C, and cells were cultivated at 5,000–7,500 cells/cm^2^ prior incubated in humid atmosphere at 37°C containing 5% carbon dioxide (CO_2_). For each passage, the number of cell divisions was calculated as log (*N*/*n*)/log 2, where *N* is the number of cells at the time of passage and *n* is the number of cells initially plated. The cells were divided into two groups which were young cells with population doubling (PD) 14 and senescent cells with PD 21.

### 2.2. Gingerol and Shogaol Preparation and Treatment Protocol

Ginger (*Zingiber officinale* Roscoe) extract was obtained from Universiti Teknologi Malaysia (UTM, Malaysia). Two types of the extraction method were used by using subcritical water extraction to extract the standardised ginger extracts [22]. For ginger extract 1 (GE1), the optimum condition was at 130°C, for 30 minutes, while the solvent to solid ratio was 28/2 ml/mg, while for ginger extract 2 (GE2), the optimum condition was at 120°C, for 20 minutes, and the solvent to solid ratio was 28/2 ml/mg. GE1 contains 6-gingerol and 6-shogaol at concentrations of 289.531 *μ*g/mL and 15.466 *μ*g/mL, respectively. Meanwhile, GE2 contains lower gingerol and higher shogaol as compared to GE1 which are 181.257 *μ*g/mL and 63.425 *μ*g/mL, respectively.

Stock solutions for GE1 and GE2 were freshly prepared in water at a concentration of 10 mg/ml. The stock solutions were kept not more than one month at −20°C. Both ginger extracts were diluted with CCM into series of concentrations, 0, 10, 20, 30, 50, 100, 200, 300, 500, and 1,000 *μ*g/mL. Myoblast cells were plated at density of 2 × 10^4^ in the 96-well plate before incubated overnight. The medium was then changed with the new medium containing different concentrations (50, 100, 200, and 300 *μ*g/mL) of GE1 and GE2 and incubated in humidified atmosphere with 5% CO_2_ at 37°C for 24 h. After 24 h of incubation, the media containing either GE1 or GE2 were replaced with the new CCM. The final concentration used in this study was taken from the previous study on the cell viability assay (manuscript under review).

### 2.3. Analysis of Cellular Morphology and Myogenic Purity

The myogenic purity was evaluated by desmin staining. Desmin is a cytoskeletal protein that is only expressed in myogenic cells and not in fibroblast cells. Myogenic purity was determined by performing immunocytochemistry analysis using the specific antibody for desmin (D33; DAKO Denmark) at a dilution of 1 : 50. Myoblasts were plated at a density of 1 × 10^4^ on *μ*-Slide 8 well (ibidi, Martinsried, Germany). The cells were then fixed with cold ethanol for 5 minutes. Then, cells were incubated sequentially with the antidesmin antibody and Alexa Flour 488 goat antimouse. Nuclei were visualized using Hoechst 33342 (Life Technologies, Carlsbad, USA). The slides were observed under an EVOS FL Digital Inverted Fluorescence Microscope (Life Technologies, Carlsbad, CA, USA). The percentage of desmin-positive cells was determined by counting at least 100 cells in three independent cultures. The morphological changes of myoblast cells were also observed.

### 2.4. SA-*β*-Galactosidase Analysis

The presence of the senescent myoblast was confirmed by evaluating the expression of SA-*β*-galactosidase (SA-*β*-gal) as described by Dimri et al. [26]. SA-*β*-gal was analysed by using Senescent Cell Histochemical Staining Kit (Sigma-Aldrich, St. Louis, Missouri, USA). Before analysis, cells were incubated in staining solution in the absence of CO_2_ at 37°C for 8 h. At least 100 cells were observed to obtain the percentage of blue-stained cells. The morphological changes of the myoblast were also evaluated.

### 2.5. Induction of Differentiation

To induce differentiation, the cells were plated at 20,000 cells/cm^2^ before incubated overnight at 37°C with 5% CO_2_. On the following day, the growth medium was replaced with DMEM: F12 (Lonza, Walkersville, MD, USA) which was supplemented with 2% horse serum (ATCC, Baltimore, USA). The cells were then treated with different concentrations of GE1 and GE2 containing 6-gingerol and 6-shogaol. Muscle cell differentiation was induced by replacing proliferation medium SkBM with a differentiation medium DMEM: F12 (Lonza, Walkersville, MD, USA), supplemented with 2% horse serum (ATCC, Baltimore, USA). The medium was changed every two days.

### 2.6. Determination of Myogenic Differentiation

Myoblast regenerative capacity was determined by myotube size, fusion index, and maturation index which indicate efficacy of myogenic differentiation. Myogenic differentiation was observed on days 1, 3, 5, and 7 of differentiation. Differentiated myoblast cells on days 0, 1, 3, 5, and 7 were cultured and stained using immunocytochemistry technique. Fusion index, maturation index, and myotube size were determined by using ImageJ software version 1.50i. The myotube size was analysed by the number of nuclei per myotube in a minimum of 11 multinucleated cells in 3 different randomly chosen optical fields [23]. The fusion index [24] and maturation index [25] were calculated in a minimum of 50 nuclei in 3 different randomly chosen optical fields, and the formula is shown as follows:

(1) $$\begin{array}{c} \text{fusion index}=\frac{\text{the number of nuclei in myotube }\left(\geq \;2\;\text{nuclei}\right)}{\text{the total number of desmin-positive nuclei }}\times 100\%,\\ \text{maturation index}=\frac{\text{the number of nuclei in myotubes }\left(\geq 5\;\text{nuclei}\right)}{\text{the total number of desmin-positive nuclei }}\;\times \;100\%. \end{array}$$

### 2.7. Cell Cycle Analysis

Ginger-treated myoblasts and untreated control cells were subcultured in the 10 cm^2^ tissue culture dish. Cells were harvested and prepared for cell cycle analysis using Cycletest PLUS DNA Reagent Kit (Becton Dickinson, USA) after 24 h of incubation. The cell cycle was analysed by using the FACS Calibur flow cytometer (Becton Dickinson, USA) using specific fluorescent dye probe, propidium iodide (PI).

### 2.8. Statistical Analysis

Each experiment was conducted at least three times, and data were presented as mean ± SD. The data were analysed by one-way analysis of variance (ANOVA) using SPSS software version 20. *p* < 0.05 was accepted as the significant value.

## 3. Results

### 3.1. Analysis of Myogenic Purity of the Myoblast

Cellular morphology and myogenic purity were observed in all groups of cells (control and treated groups) by determining desmin expression. The myogenic purity of the young and senescent myoblast was more than 95% in both control and ginger-treated myoblasts (Tables 1 and 2).

### 3.2. Effect of Ginger on Cellular Morphology of the Human Myoblast

**Figure 1:**
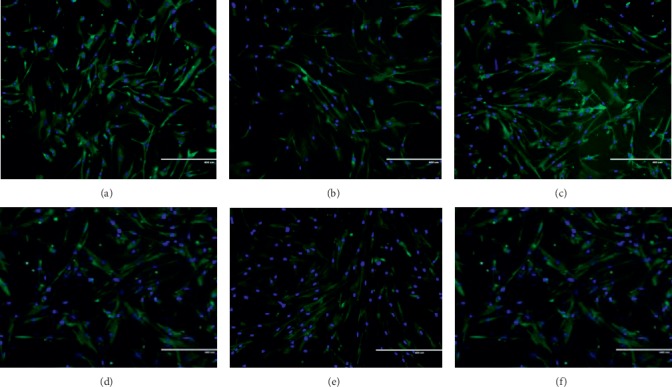
Effects of GE1 treatment on the cellular morphology of myoblasts. The micrographs of myoblasts were taken from the young control (a), 50 *μ*g/mL GE1-treated young (b), 200 *μ*g/mL GE1-treated young (c), control senescent (d), 100 *μ*g/mL GE1-treated senescent (e), and 300 *μ*g/mL GE1-treated senescent (f). Myoblasts were stained with green colour for desmin and blue colour for nuclei.

**Figure 2:**
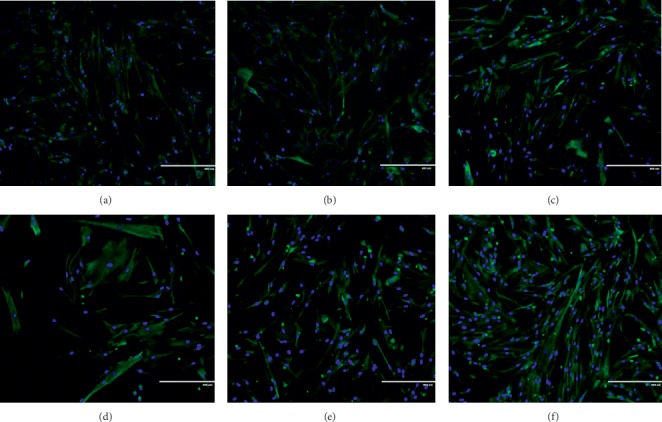
Effects of GE2 treatment on the cellular morphology of myoblasts. The micrographs of myoblasts were taken from the young control (a), 50 *μ*g/mL GE2-treated young (b), 200 *μ*g/mL GE2-treated young (c), control senescent (d), 100 *μ*g/mL GE2-treated senescent (e), and 300 *μ*g/mL GE2-treated senescent (f). Myoblast was stained with green colour for desmin and blue colour for nuclei.

Control young myoblast displayed a normal spindle shape with round nuclei and lacked striation (Figures 1(a) and 2(a)). However, during cellular senescence, the myoblast became larger and flatter with prominent filaments in the cytoplasm (Figures 1(d) and 2(d)). After treated with GE1 (Figures 1(e) and 1(f)) and GE2 (Figures 2(e) and 2(f)), the senescent myoblast resembled young myoblast morphology by showing normal spindle shape and round nuclei. Senescent myoblasts treated with high concentration (300 *μ*g/mL) of GE1 and GE2 displayed more normal spindle shape and round nuclei as compared with low concentration (50 *μ*g/mL) of GE1 and GE2.

### 3.3. Effects of Ginger on Cellular Senescence of the Human Myoblast (SA-*β*-Galactosidase Analysis)

**Figure 3:**
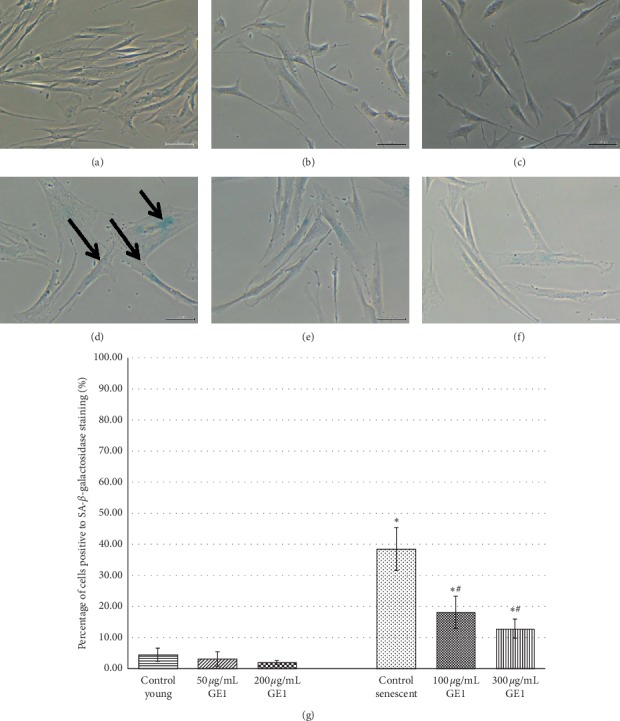
Effects of GE1 treatment on senescent biomarker SA-*β*-galactosidase. The micrographs of the myoblast were captured from control young (a), 50 *μ*g/mL GE1-treated young (b), 200 *μ*g/mL GE1-treated young (c), control senescent (d), 100 *μ*g/mL GE1-treated senescent (e), and 300 *μ*g/mL GE1-treated senescent (f) cells. As shown by the arrow in (d), the senescent control cells were stained blue indicating the positive result for SA-*β*-gal expression. The percentage of cells positive to SA-*β*-gal staining was measured quantitatively (g). The cells positively stained for SA-*β*-gal were significantly reduced with GE1 treatment. ^*∗*^*p* < 0.05 Significantly different as compared with control young myoblasts; ^#^*p* < 0.05 significantly different as compared with control senescent myoblasts. The data were presented as the mean ± SD, *n* = 9.

**Figure 4:**
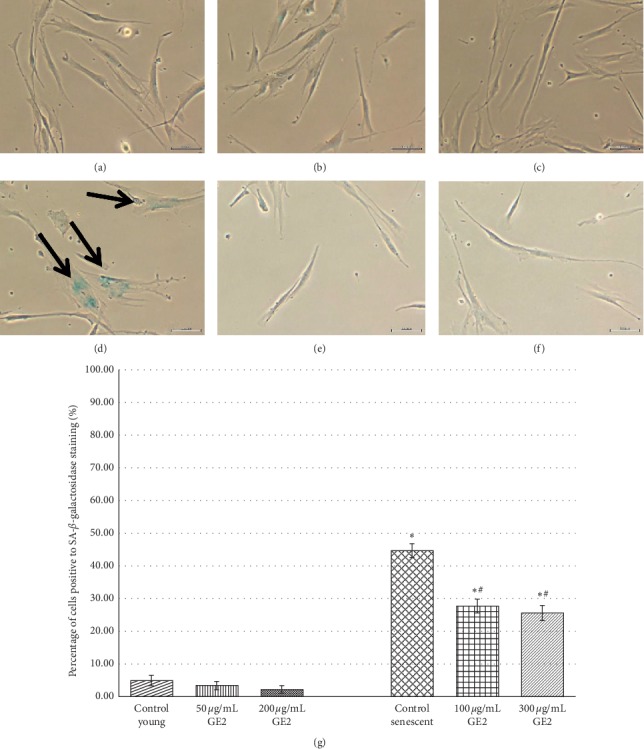
Effects of GE2 treatment on senescent biomarker SA-*β*-galactosidase. The micrographs of myoblasts were captured from control young (a), 50 *μ*g/mL GE2-treated young (b), 200 *μ*g/mL GE2-treated young (c), control senescent (d), 100 *μ*g/mL GE2-treated senescent (e), and 300 *μ*g/mL GE2-treated senescent (f) cells. As shown by the arrow in (d), the senescent control was stained blue indicating the positive result for SA-*β*-gal. The percentage of cells positive to SA-*β*-gal staining was measured quantitatively (g). The cells positively stained for SA-*β*-gal were significantly reduced with GE2 treatment ^*∗*^*p* < 0.05 Significantly different as compared with control young myoblasts; ^#^*p* < 0.05 significantly different as compared with control senescent myoblasts. The data were presented as the mean ± SD, *n* = 9.

SA-*β*-gal analysis was performed by determining the presence of blue stain which indicates the senescence-associated biomarker in myoblasts. Positively stained senescent myoblasts are shown in Figures 3(d)–3(f) and 4(d)–4(f), while young myoblasts are shown in Figures 3(a)–3(c) and 4(a)–4(c). The percentage of SA-*β*-gal-positive cells for GE1 and GE2 treatment in young and senescent myoblast cells is shown in Figures 3(g) and 4(g). There was a significant increase in SA-*β*-gal expression in the senescent myoblast as compared to young cells (*p* < 0.05). However, the percentage of SA-*β*-gal-positive cells in the senescent myoblast was significantly decreased with GE1 and GE2 treatment as compared to untreated control cells (*p* < 0.05). Both concentrations of GE1 and GE2 treatment caused a significant reduction in SA-*β*-gal expression in senescent myoblasts (*p* < 0.05).

### 3.4. Effect of Ginger on Myoblast Regenerative Capacity

**Figure 5:**
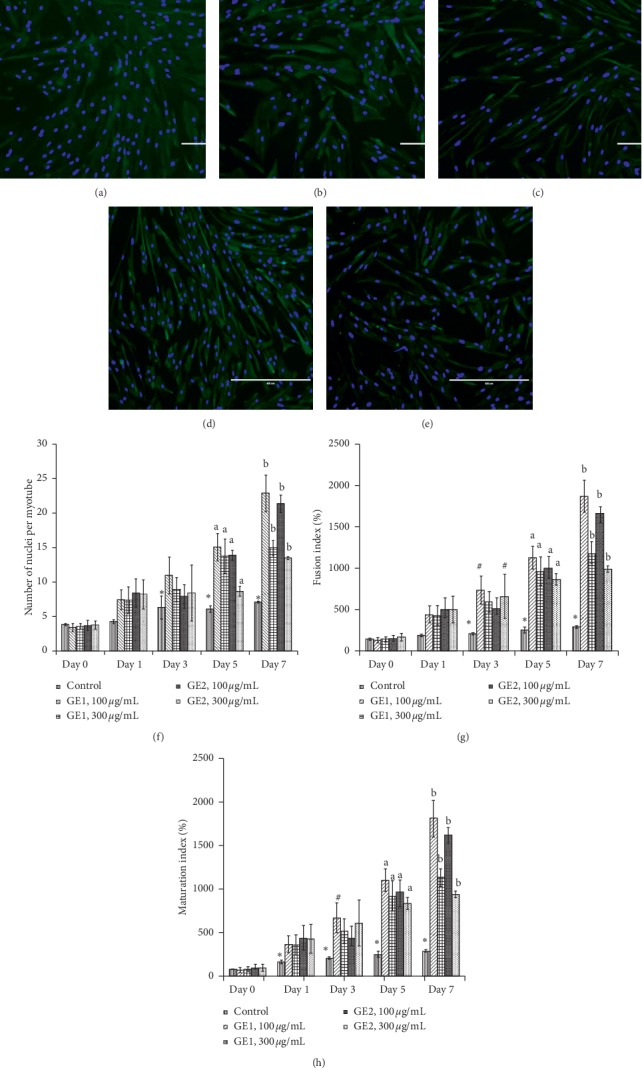
Effects of ginger (*Zingiber officinale* Roscoe) on myoblast differentiation. The micrographs of myoblasts were taken from the control (a), 100 *μ*g/mL GE1-treated myoblast (b), 300 *μ*g/mL GE1-treated myoblast (c), 100 *μ*g/mL GE2-treated myoblast (d), and 300 μg/mL GE2-treated myoblast (e) on day 7 of differentiation. Myoblast was stained with green colour for desmin and blue colour for nuclei. The myotube sizes (f), fusion index (g), and maturation index (h) were quantitated on day 0, day 1, day 3, day 5, and day 7 of differentiation for different concentrations (100 *μ*g/mL and 300 *μ*g/mL) of GE1 and GE2 treatment in senescent myoblast cells. Data are presented as means ± SD, *n* = 3. ^*∗*^*p* < 0.05: significantly different compared with the control group (day 0), ^#^*p* < 0.05: significantly different compared with the control (day 3), ^a^*p* < 0.05: significantly different compared with the control (day 5), and ^b^*p* < 0.05: significantly different compared with the control (day 7).

Effect of ginger on myoblast regenerative capacity was determined based on myotube size, fusion index, and maturation index. Figures 5(a)–5(e) show the positive desmin stained that represents myoblast differentiation for each treatment group on day 7 of differentiation. The myotube size, percentage of fusion index, and percentage of maturation index of the myoblast on different days of differentiation are shown in Figures 5(f)–5(h) and in Table 3 (A)–(C).

The number of nuclei per myotube in the control myoblast was significantly higher on days 3, 5, and 7 of differentiation as compared with day 0 (*p* < 0.05) (Figure 5(f)and Table 3 (A)), while on day 5, there was a significant increase in myotube size for the 100 *μ*g/mL GE1-treated myoblast, 300 *μ*g/mL GE1-treated myoblast, 100 *μ*g/mL GE2-treated myoblast, and 300 *μ*g/mL GE2-treated myoblast as compared with the control (*p* < 0.05). On day 7 of myoblast differentiation, the number of nuclei per myotube was significantly higher for 100 *μ*g/mL GE1, 300 *μ*g/mL GE1, 100 *μ*g/mL GE2, and 300 *μ*g/mL GE2 (*p* < 0.05).

Meanwhile, the fusion index of the control myoblast was significantly higher on days 3, 5, and 7 of differentiation as compared with day 0 (*p* < 0.05) (Figure 5(g) and Table 3 (B)). Treatment with 100 *μ*g/mL GE1 and 300 *μ*g/mL GE2 on day 3 significantly increased the fusion index of the myoblast as compared with the control (*p* < 0.05). Meanwhile, the fusion index on days 5 and 7 was significantly increased for concentration of 100 *μ*g/mL GE1, 300 *μ*g/mL GE1, 100 *μ*g/mL GE2, and 300 *μ*g/mL GE2 as compared with the control (*p* < 0.05).

The maturation index of control myoblasts was significantly higher on days 1, 3, 5, and 7 of differentiation compared with day 0 (*p* < 0.05) (Figure 5(h) and Table 3 (C)). On day 3, treatment with 100 *μ*g/mL of GE1 caused a significant increase to the maturation index as compared with the control group of the myoblast (*p* < 0.05). Treatment with GE1 at concentrations of 100 *μ*g/mL and 300 *μ*g/mL significantly increased the maturation index of myoblasts as compared with control myoblasts on days 5 and 7 of differentiation (*p* < 0.05). Meanwhile, for treatment with GE2, there was also a significant increase in the maturation index at concentrations of 100 *μ*g/mL and 300 *μ*g/mL of GE2 on days 5 and 7 compared with the control group (*p* < 0.05).

### 3.5. Effect of Ginger on Cell Cycle Profile

**Figure 6:**
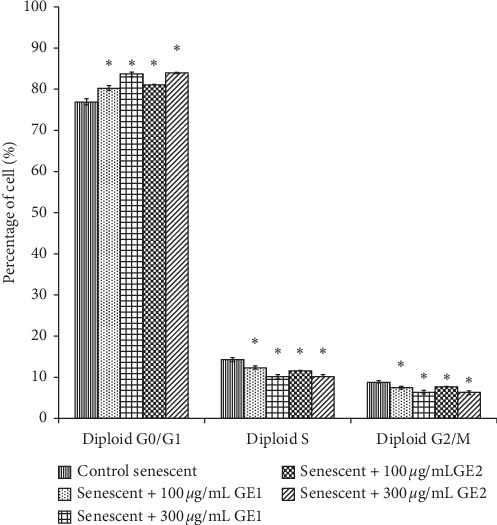
Cell cycle profile of the senescent myoblast treated with different concentrations of ginger (*Zingiber officinale* Roscoe) extracts 1 and 2. ^*∗*^*p* < 0.05 compared to control senescent. The data were presented as the mean ± SD, *N* = 3.

Treatment with GE1 and GE2 caused a significant increase in the G_0_/G_1_ phase of senescent myoblasts as compared with control senescent myoblasts (*p* < 0.05) (Figure 6). The percentage of cells in phase S, however, was significantly reduced with GE1 and GE2 treatment as compared with the untreated control (*p* < 0.05). Treatment with GE1 and GE2 also caused a significant reduction in the percentage of cells in the G_2_/M phase as compared with the untreated control (*p* < 0.05).

## 4. Discussion

Myoblast is an embryonic progenitor cell originated from differentiation process of satellite cells. It is essential for the formation of muscle tissue. From the satellite cell, myoblasts will further differentiate into myotube which is depicted by the presence of multiple nuclei in its fibres [27]. Normally, young myoblasts will differentiate into large-branched myotube, and senescent myoblasts will turn into smaller myotube [23]. The morphology of the young myoblast is normally thin, elongated, and spindle shaped, while senescent of the myoblast turned into a flattened and broader structure with larger cytoplasm.

Previous study reported that the senescent myoblast displayed high level of reactive oxygen species (ROS), downregulation of myogenic differentiation, low proliferation capacity, and high expression of oxidative damage-associated genes [23, 28]. The high level of ROS contributes to the increment of oxidative stress level that causes oxidative damage to protein and DNA, as well as it increases lipid peroxidation which later contributes to mitochondrial dysfunction, alters cell growth, and induces mutation as well as apoptosis [7, 8]. Huang et al. reported that the accumulation of ROS during senescent leads to the alteration of cellular metabolism and pathway which contributes to the changes in the cell membrane, cytoskeleton, and extracellular matrix (ECM) which are vital in maintaining the integrity of the myoblast cell structure [29]. Therefore, the morphology of the senescent myoblast is different as compared with the morphology of the young myoblast.

Ginger (*Zingiber officinale* Roscoe) is one of the potential herbs that can be introduced in order to reverse muscle ageing by exhibiting its antioxidant properties. There are two main active compounds that contribute to the therapeutic effects in ginger which are 6-gingerol and 6-shogaol. 6-Shogaol is the dehydrated form of 6-gingerol which is formed when fresh ginger is dried or treated with high temperature [30]. These bioactive compounds displayed an antioxidant property via the nuclear factor erythroid 2-related factor (Nrf2) signalling pathway [31]. The introduction of ginger into the cell leads to the dissociation of Nrf2 from Kelch-like ECH-associated protein 1 (Keap1), an inhibitory protein partner in cytosol which results in the translocation of Nrf2 into the nucleus [32]. These Nrf2 will bind to the antioxidant response element (ARE) and induce the transcription of antioxidant genes exhibiting the protective effect of ginger.

In this study, the effects of 6-gingerol and 6-shogaol in two types of ginger standardised extract on myoblast cells in culture were elucidated. The composition of 6-gingerol and 6-shogaol in these two ginger extracts was different. GE1 consists of 289.531 mg/mL of 6-gingerol and 15.466 mg/mL of 6-shogoal, while GE2 consists of 181.257 mg/mL of 6-gingerol and 63.425 mg/mL of 6-shogoal. Several factors contribute to these differences such as the type of extraction, temperature, pressure, time, and solvent used in the extraction process [33]. In this study, two different extraction conditions were applied in preparing the extracts. GE1 was extracted by using higher temperature with prolonged time of extraction as compared to GE2 resulting in higher concentration of 6-gingerol and 6-shogoal. A previous study showed that the quantity of ginger oil extraction was decreased with the increasing of temperature and changes in pressure [33]. This difference may be due to the difference in the extraction method of ginger.

The findings of our study showed that the morphology of senescent myoblasts treated with standardised ginger extracts containing 6-gingerol and 6-shogaol resembled the morphology of young myoblasts. 6-Gingerol- and 6-shogaol-treated senescent myoblast displayed a round nuclei and normal spindle shape of the young myoblast. This result was in accordance with the study performed by Khor et al., which found that the senescent myoblast treated with tocotrienol-rich fraction (TRF) displayed a similar morphology as the young myoblast cell [23]. The changes of morphology in the senescent myoblast treated with 6-gingerol and 6-shogaol may be due to the modulation of protein expression of matrix metalloproteinases (MPPs). It has been reported that higher level of ROS during senescence contributes to the activation of MAP kinase, which is vital in the transcriptional regulation of MMPs [34]. These MMPs are responsible to degrade procollagen in the myoblast cell as well as regulate skeletal muscle cell migration, differentiation, and regeneration which influence the structure of the myoblast [35]. A previous study reported that 6-gingerol and 6-shagoal inhibited the expression of MMPs which is highly expressed during senescence by inhibiting the MAP kinase and PI3k/Akt pathway as well as NF-*β* and STAT3 activities [36]. The inhibition of MMP expression will then reduce the changes in morphology of the senescent myoblast.

One of the most important features of senescence is the expression of senescence-associated *β*-galactosidase (SA-*β*-gal) which acts as a replicative senescent biomarker. Our study demonstrated that treatment with 6-gingerol and 6-shogaol reduced the level of SA-*β*-gal in the senescent myoblast. This proved that 6-gingerol and 6-shogaol were able to exhibit ageing reversal effect on myoblast cells in the culture. This finding is in agreement with the finding from a previous study which reported that treatment with TRF and alpha-tocopherol (ATF) on the senescent myoblast [23] and H_2_O_2_-induced myoblast [37] caused a decrease in SA-*β*-gal expression. Our current study also revealed that GE1 which contains higher amount of 6-gingerol with less 6-shogoal exhibited similar reduction in SA-*β*-gal expression as compared with the effects of GE2 which contains higher level of 6-shogoal with lower 6-gingerol. Even though both 6-gingerol and 6-shogaol possess different chemical structures, they produced similar effects on myoblasts in the culture when given in combination with the standardised ginger extracts. A previous study reported that based on the chemical structure of 6-shogaol, there is conjugation of *α*, *β*-unsaturated ketone skeleton which may contribute to the positive effect of 6-shogaol [38]. Another study reported that 6-shogaol exerted better potency and efficacy as compared to 6-gingerol as an anticancer, anti-inflammatory, and antioxidant [38].

The ability of the myoblast cell to differentiate was confirmed by measuring the number of nuclei per myotube, fusion indexes, and maturation indexes. The differentiated myoblast can be seen through formation of the multinucleated myoblast. Our findings showed that the senescent myoblast underwent differentiation as it caused increment in fusion indexes, maturation indexes, and the number of nuclei per myotube. Treatment with different concentrations of ginger extract was found to improve differentiation of the senescent myoblast by increasing the fusion indexes, maturation indexes, and myotube size. This result was similar to our previous findings which found that treatment with *Chlorella vulgaris* on the myoblast has promoted the differentiation in the young myoblast by improving myotube size, fusion index, and maturation index [39]. In another study, treatment with TRF on the myoblast resulted in increased fusion index and myotube surface area in both young and senescent myoblasts [40]. Meanwhile, ATF treatment on the senescent myoblast displayed a significant decrease in the fusion index and size of myotubes which contradicted with ginger extract treatment on the senescent myoblast [23]. This finding indicated the potential of ginger extract in promoting myoblast differentiation and regeneration in the senescent myoblast.

Our results also showed that treatment with 6-gingerol and 6-shogaol affects the progression of cell cycle of myoblast cells. Normally, myoblasts undergo active proliferation before entering the differentiation phase which consists of cell cycle arrest and fusion of the myoblast cell into the myotube [41]. In the G_0_/G_1_ phase, cells are growth-arrested as prevention for replication to allow repair of damaged DNA [42]. During this phase, DNA damage and other factors are being assessed, and if there are inadequate conditions, the cell will not be allowed to enter the next phase of the cell cycle. A previous study found that the expression of the Ki67 protein which is essential in cell proliferation was low during the G_0_/G_1_ phase resulting in loss of proliferative capacity in the myoblast cell [43]. Previous finding also reported that the expression of the Ki67 protein was decreased in the senescent myoblast as compared with the young myoblast leading to the decline in cellular proliferation of the senescent myoblast [44]. Moreover, cellular proliferation is influenced by telomere length and telomerase activity. As ageing occurs, telomere length and telomerase activity are decreased resulting in the capacity reduction of cells to proliferate [45]. Treatment with the natural compound such as TRF on senescent fibroblast cells has been reported to stimulate the expression of telomerase and increased elongation of telomere resulting in cells commit to the S-phase [46]. 6-Gingerol and 6-shogaol may have a similar effect on fibroblast cells but not on myoblasts. Razak et al. demonstrated in their findings that, during the early stage of differentiation, there was promotion of cell cycle withdrawal to facilitate myoblast differentiation [40]. This was further confirmed in the findings of this study which showed decreased senescent myoblast cell population in the S-phase suggesting cell proliferation was inhibited in preparation for cell differentiation. The findings observed in this study strengthen the facts that 6-gingerol and 6-shogoal may protect against cellular senescence and promote myoblast differentiation.

## 5. Conclusion

In conclusion, standardised extract of ginger which consists of 6-gingerol and 6-shogaol has great potential in preventing cellular senescence of human myoblasts and promotes myoblast differentiation. Both 6-gingerol and 6-shogaol exerted a comparable effect in preventing cellular senescence of human myoblasts which also promote myoblast differentiation. This may give an advantage in the prevention of muscle disease particularly sarcopenia.

## Figures and Tables

**Table 1:** Percentage of desmin-positive cells for GE1 treatment in young and senescent myoblast cells.

Myoblasts	Young			Senescent		

Concentration of ginger extracts (*μ*g/mL)	Control	50	200	Control	50	300
Desmin-positive cell (%)	97.32	98.93	98.36	97.34	96.24	96.49

**Table 2:** Percentage of desmin-positive cells for GE2 treatment in young and senescent myoblast cells.

Myoblasts	Young			Senescent		

Concentration of ginger extracts (*μ*g/mL)	Control	50	200	Control	50	300
Desmin-positive cell (%)	98.70	96.85	95.48	96.67	97.34	97.40

**Table 3:** Myotube size (A), fusion index (B), and maturation index (C) for GE1 and GE2 treatment in senescent myoblast cells.

	Day 0	Day 1	Day 3	Day 5	Day 7


*(A) Myotube size (number of nuclei per myotube)*					
Control	3.83 ± 0.17	4.25 ± 0.27	6.30 ± 1.65	6.07 ± 0.47	7.07 ± 0.12
100 *μ*g/mL GE1-treated myoblast	3.40 ± 0.57	7.44 ± 1.40	10.96 ± 2.65	15.06 ± 1.99	22.87 ± 2.65
300 *μ*g/mL GE1-treated myoblast	3.58 ± 0.42	7.38 ± 1.92	8.94 ± 1.73	13.76 ± 2.47	14.95 ± 1.10
100 *μ*g/mL GE2-treated myoblast	3.67 ± 0.74	8.41 ± 2.04	7.89 ± 1.69	13.88 ± 0.69	21.37 ± 1.26
300 *μ*g/mL GE2-treated myoblast	3.75 ± 0.59	8.27 ± 2.10	8.383 ± 4.07	8.64 ± 0.67	13.52 ± 0.18

*(B) Fusion index (%)*					
Control	142.36 ± 10.71	188.91 ± 16.13	205.44 ± 15.48	253.49 ± 35.14	289.14 ± 14.06
100 *μ*g/mL GE1-treated myoblast	130.01 ± 33.43	439.05 ± 101.72	735.87 ± 170.41	1126.08 ± 136.36	1868.75 ± 193.04
300 *μ*g/mL GE1-treated myoblast	138.64 ± 32.28	428.48 ± 117.43	592.86 ± 125.59	962.76 ± 169.81	1173.37 ± 101.12
100 *μ*g/mL GE2-treated myoblast	146.40 ± 43.73	500.94 ± 137.12	511.81 ± 128.92	1003.54 ± 135.43	1662.94 ± 84.07
300 *μ*g/mL GE2-treated myoblast	165.05 ± 45.54	500.85 ± 159.23	659.62 ± 263.66	863.93 ± 67.28	988.85 ± 38.51

*(C) Maturation index (%)*					
Control	78.65 ± 3.02	161.27 ± 20.25	205.44 ± 15.48	248.73 ± 35.13	286.29 ± 14.64
100 *μ*g/mL GE1-treated myoblast	67.02 ± 30.21	363.65 ± 97.84	669.06 ± 170.15	1099.34 ± 129.41	1812.29 ± 209.96
300 *μ*g/mL GE1-treated myoblast	80.125 ± 24.65	356.51 ± 116.49	517.24 ± 138.36	920.32 ± 173.30	1132.88 ± 104.01
100 *μ*g/mL GE2-treated myoblast	88.42 ± 48.41	433.31 ± 147.51	438.71 ± 133.89	966.51 ± 134.50	1623.49 ± 81.31
300 *μ*g/mL GE2-treated myoblast	95.40 ± 40.82	427.50 ± 165.71	610.44 ± 265.51	835.20 ± 69.78	938.97 ± 43.51

Values are mean + SD, *N* = 3.

## Data Availability

The data used to support the findings of this study are included within the article.

## References

[1] Walston J. D. (2012). Sarcopenia in older adults. *Current Opinion in Rheumatology*.

[2] Kim T. N., Choi K. M. (2013). Sarcopenia: definition, epidemiology, and pathophysiology. *Journal of Bone Metabolism*.

[3] McCormick R., Vasilaki A. (2018). Age-related changes in skeletal muscle: changes to life-style as a therapy. *Biogerontology*.

[4] Gomes M. J., Martinez P. F., Pagan L. U. (2017). Skeletal muscle aging: influence of oxidative stress and physical exercise. *Oncotarget*.

[5] Palipoch S., Koomhin P. (2015). Oxidative stress-associated pathology: a review. *Sains Malaysiana*.

[6] Norshafarina S. K., Ibrahim M. S. N., Suzana S., Hasnan A. M., Zahara M., Zaitun Y. (2013). Sarcopenia and its impact on health: do they have significant associations?. *Sains Malaysiana*.

[7] Khansari N., Shakiba Y., Mahmoudi M. (2009). Chronic inflammation and oxidative stress as a major cause of age-related diseases and cancer. *Recent Patents on Inflammation & Allergy Drug Discovery*.

[8] Ayala A., Muñoz M. F., Argüelles S. (2014). Lipid peroxidation: production, metabolism, and signaling mechanisms of malondialdehyde and 4-hydroxy-2-nonenal. *Oxidative Medicine and Cellular Longevity*.

[9] Mošovská S., Nováková D., Kaliňák M. (2015). Antioxidant activity of ginger extract and identification of its active components. *Acta Chimica Slovaca*.

[10] Saha A., Blando J., Silver E., Beltran L., Sessler J., DiGiovanni J. (2014). 6-shogaol from dried ginger inhibits growth of prostate cancer cells both *in vitro* and *in vivo* through inhibition of STAT3 and NF-*κ*B signaling. *Cancer Prevention Research*.

[11] Park M., Bae J., Lee D.-S. (2008). Antibacterial activity of [10]-gingerol and [12]-gingerol isolated from ginger rhizome against periodontal bacteria. *Phytotherapy Research*.

[12] Al-Amin Z. M., Thomson M., Al-Qattan K. K., Peltonen-Shalaby R., Ali M. (2006). Anti-diabetic and hypolipidaemic properties of ginger (*Zingiber officinale*) in streptozotocin-induced diabetic rats. *British Journal of Nutrition*.

[13] Ezzat S. M., Ezzat M. I., Okba M. M., Menze E. T., Abdel-Naim A. B. (2018). The hidden mechanism beyond ginger (*Zingiber officinale Rosc.*) potent *in vivo* and *in vitro* anti-inflammatory activity. *Journal of Ethnopharmacology*.

[14] Tanaka K., Arita M., Sakurai H., Ono N., Tezuka Y. (2015). Analysis of chemical properties of edible and medicinal ginger by metabolomics approach. *BioMed Research International*.

[15] Wu H.-C., Horng C.-T., Tsai S.-C. (2018). Relaxant and vasoprotective effects of ginger extracts on porcine coronary arteries. *International Journal of Molecular Medicine*.

[16] Yusof Y. A. M., Abdul-Aziz A. (2005). Effects of *Zingiber officinale* on superoxide dismutase, glutathione peroxidase, catalase, glutathione and malondialdehyde content in HepG2 cell line. *Malaysian Journal of Biochemistry and Molecular Biology*.

[17] Akinyemi A. J., Ademiluyi A. O., Oboh G. (2013). Aqueous extracts of two varieties of ginger (*Zingiber officinale*) inhibit Angiotensin i–converting enzyme, iron (II), and sodium nitroprusside-induced lipid peroxidation in the rat heart *in vitro*. *Journal of Medicinal Food*.

[18] Hosseinzadeh A., Juybari K. B., Fatemi M. J. (2017). Protective effect of ginger (*Zingiber officinale roscoe*) extract against oxidative stress and mitochondrial apoptosis induced by interleukin-1*β* in cultured chondrocytes. *Cells Tissues Organs*.

[19] Lee C., Park G. H., Kim C.-Y., Jang J.-H. (2011). [6]-gingerol attenuates beta-amyloid-induced oxidative cell death via fortifying cellular antioxidant defense system. *Food and Chemical Toxicology*.

[20] Park G., Kim H. G., Ju M. S. (2013). 6-shogaol, an active compound of ginger, protects dopaminergic neurons in Parkinson’s disease models via anti-neuroinflammation. *Acta Pharmacologica Sinica*.

[21] Moon M., Kim H. G., Choi J. G. (2014). 6-Shogaol, an active constituent of ginger, attenuates neuroinflammation and cognitive deficits in animal models of dementia. *Biochemical and Biophysical Research Communications*.

[22] Sarip M. (2012). Subcritical water extraction of 6-gingerol and 6-shogaol from *Zingiber officinale*.

[23] Khor S. C., Razak A. M., Ngah W. Z. W., Yusof Y. A. M., Karim N. A., Makpol S. (2016). The tocotrienol-rich fraction is superior to tocopherol in promoting myogenic differentiation in the prevention of replicative senescence of myoblasts. *PLoS One*.

[24] Bigot A., Jacquemin V., Debacq-Chainiaux F. (2008). Replicative aging down-regulates the myogenic regulatory factors in human myoblasts. *Biology of the Cell*.

[25] Sun Y., Ge Y., Drnevich J., Zhao Y., Band M., Chen J. (2010). Mammalian target of rapamycin regulates miRNA-1 and follistatin in skeletal myogenesis. *The Journal of Cell Biology*.

[26] Dimri G. P., Lee X., Basile G. (1995). A biomarker that identifies senescent human cells in culture and in aging skin *in vivo*. *Proceedings of the National Academy of Sciences*.

[27] Yin H., Price F., Rudnicki M. A. (2013). Satellite cells and the muscle stem cell niche. *Physiological Reviews*.

[28] Pääsuke R., Eimre M., Piirsoo A. (2016). Proliferation of human primary myoblasts is associated with altered energy metabolism in dependence on ageing *in vivo* and *in vitro*. *Oxidative Medicine and Cellular Longevity*.

[29] Huang X., Chen L., Wanjing L. (2015). Involvement of oxidative stress and cytoskeletal disruption in microcystin-induced apoptosis in CIK cells. *Aquatic Toxicology*.

[30] Jolad S. D., Lantz R. C., Solyom A. M., Chen G. J., Bates R. B., Timmermann B. N. (2004). Fresh organically grown ginger (*Zingiber officinale*): composition and effects on LPS-induced PGE_2_ production. *Phytochemistry*.

[31] Peng S., Yao J., Liu Y., Duan D., Zhang X., Fang J. (2015). Activation of Nrf2 target enzymes conferring protection against oxidative stress in PC12 cells by ginger principal constituent 6-shogaol. *Food & Function*.

[32] Lee J.-M., Li J., Johnson D. A. (2005). Nrf2, a multi-organ protector?. *The FASEB Journal*.

[33] Kanadea R., Bhatkhandeb D. (2016). Extraction of ginger oil using different methods and effect of solvents, time, temperature to maximize yield. *International Journal of Advances in Science Engineering and Technology*.

[34] Freitas-Rodriguez S., Folgueras A. R., Lopez-Otin C. (2017). The role of matrix metalloproteinases in aging: tissue remodeling and beyond. *Biochimica et Biophysica Acta (BBA)-Molecular Cell Research*.

[35] Hiyama A., Sakai D., Risbud M. V. (2010). Enhancement of intervertebral disc cell senescence by WNT/*β*-catenin signaling-induced matrix metalloproteinase expression. *Arthritis & Rheumatism*.

[36] Weng C.-J., Chou C.-P., Ho C.-T., Yen G.-C. (2012). Molecular mechanism inhibiting human hepatocarcinoma cell invasion by 6-shogaol and 6-gingerol. *Molecular Nutrition & Food Research*.

[37] Lim J. J., Ngah W. Z. W., Mouly V., Karim N. A. (2013). Reversal of myoblast aging by tocotrienol rich fraction posttreatment. *Oxidative Medicine and Cellular Longevity*.

[38] Kou X., Wang X., Ji R. (2018). Occurrence, biological activity and metabolism of 6-shogaol. *Food & Function*.

[39] Azlan N. Z., Yusof Y. A. M., Alias E., Makpol S. (2019). *Chlorella vulgaris* improves the regenerative capacity of young and senescent myoblasts and promotes muscle regeneration. *Oxidative Medicine and Cellular Longevity*.

[40] Razak A. M., Khor S. C., Jaafar F., Karim N. A., Makpol S. (2018). Targeting myomiRs by tocotrienol-rich fraction to promote myoblast differentiation. *Genes & Nutrition*.

[41] Mackey A. L., Kjaer M., Charifi N. (2009). Assessment of satellite cell number and activity status in human skeletal muscle biopsies. *Muscle & Nerve*.

[42] Walsh K., Perlman H. (1997). Cell cycle exit upon myogenic differentiation. *Current Opinion in Genetics & Development*.

[43] Scholzen T., Gerdes J. (2000). The Ki-67 protein: from the known and the unknown. *Journal of Cellular Physiology*.

[44] Hu Z., Klein J. D., Mitch W. E., Zhang L., Martinez I., Wang X. H. (2014). MicroRNA-29 induces cellular senescence in aging muscle through multiple signaling pathways. *Aging*.

[45] Zhu C. H., Mouly V., Cooper R. N. (2007). Cellular senescence in human myoblasts is overcome by human telomerase reverse transcriptase and cyclin-dependent kinase 4: consequences in aging muscle and therapeutic strategies for muscular dystrophies. *Aging Cell*.

[46] Makpol S., Durani L. W., Chua K. H., Yusof Y. A. M., Ngah W. Z. W. (2011). Tocotrienol-rich fraction prevents cell cycle arrest and elongates telomere length in senescent human diploid fibroblasts. *Journal of Biomedicine and Biotechnology*.

